# Longitudinal imaging of T cell-based immunotherapy with multi-spectral, multi-scale optoacoustic tomography

**DOI:** 10.1038/s41598-020-61191-z

**Published:** 2020-03-17

**Authors:** Melanie A. Kimm, Stratis Tzoumas, Sarah Glasl, Murad Omar, Panagiotis Symvoulidis, Ivan Olefir, Ernst J. Rummeny, Reinhard Meier, Vasilis Ntziachristos

**Affiliations:** 1 Department of Diagnostic and Interventional Radiology, School of Medicine & Klinikum rechts der Isar, Technical University of Munich, Munich, Germany; 2 Chair for Biological Imaging, School of Medicine & Klinikum rechts der Isar, Technical University of Munich, Munich, Germany; 3 Institute of Biological and Medical Imaging, Helmholtz Zentrum München, Neuherberg, Germany

**Keywords:** Cellular imaging, Imaging the immune system

## Abstract

Most imaging studies of immunotherapy have focused on tracking labeled T cell biodistribution *in vivo* for understanding trafficking and homing parameters and predicting therapeutic efficacy by the presence of transferred T cells at or in the tumour mass. Conversely, we investigate here a novel concept for longitudinally elucidating anatomical and pathophysiological changes of solid tumours after adoptive T cell transfer in a preclinical set up, using previously unexplored in-tandem macroscopic and mesoscopic optoacoustic (photoacoustic) imaging. We show non-invasive *in vivo* observations of vessel collapse during tumour rejection across entire tumours and observe for the first time longitudinal tumour rejection in a label-free manner based on optical absorption changes in the tumour mass due to cellular decline. We complement these observations with high resolution episcopic fluorescence imaging of T cell biodistribution using optimized T cell labeling based on two near-infrared dyes targeting the cell membrane and the cytoplasm. We discuss how optoacoustic macroscopy and mesoscopy offer unique contrast and immunotherapy insights, allowing label-free and longitudinal observations of tumour therapy. The results demonstrate optoacoustic imaging as an invaluable tool in understanding and optimizing T cell therapy.

## Introduction

Immunotherapies are emerging as a potent cancer treatment approach^1^. Clinical studies using autologous, *ex vivo* stimulated cytotoxic T lymphocytes have demonstrated therapeutic efficacy^2–4^. To achieve therapeutic effects and increase patient survival, it is necessary that the transferred T cells survive, home to and enter the tumour mass, recognize antigen peptides expressed by tumour cells and induce tumour rejection. Nevertheless, endogenous cancer mechanisms often prevent tumour cell killing by T cells even though the cells had shown specificity and functionality *in vitro*. Cancer immunoediting^5^ and editing the tumour microenvironment^6^ are major drawbacks in establishing successful T cell therapies.

Imaging has been employed in the context of cellular based therapies for better understanding T cell trafficking and biodistribution^7–10^, for obtaining a mechanistic understanding of tumour elimination due to the CTL activity and for assessing therapeutic efficacy. Radiological and optical imaging methods such as PET^11^, SPECT-CT^12^, MRI^9^, bioluminescence^13^ and fluorescence imaging^14^ have been employed for visualizing labeled T cell biodistribution at different stages of therapy; a goal that has been generally achieved revealing T cell traffic and homing dynamics. Nevertheless, T cell homing and accumulation within the tumour does not always correlate with therapeutic efficacy^7,15,16^. Therefore, imaging studies that solely focus on T cell biodistribution may be limited in predicting therapeutic efficiency or revealing the process and mechanisms of tumour rejection.

Mouse imaging has been considered to optimize cytotoxic therapy by understanding efficacy and cell dynamics. In addition to macroscopic observations, intra-vital microscopy has enabled critical insights into the mechanisms behind T cell therapies by visualizing in high-resolution single cell motility and cell-cell interactions *in vivo*^7,14,17^. Two-photon microscopy has revealed the primary role of CTLs as cytotoxic effectors in the process of tumour rejection^8^ and the key role of antigen expression in T cell motility. Moreover, confocal microscopy has recently showcased the visualization of T cell biodistribution over-time in high-resolution and revealed the important role of vasculature and vessel regression during immunotherapy^17^. Vessel normalization has been shown to be a critical aspect in delivering therapeutics as well as therapeutic cells into the tumour mass to allow tumour destruction^18^. Despite its essential role in discovery, intra-vital microscopy has a limited penetration depth and field of view and therefore cannot observe events occurring macroscopically throughout the entire tumour mass. Moreover, the complex imaging protocols often requiring surgical implantation of transparent windows limit widespread dissemination.

In this work, we aimed to retrieve multi-scale information on therapeutic effects following T cell therapy, using optoacoustic imaging at the macro- and mesoscopic level. The study aimed to explore optoacoustic imaging as a novel method to understand immunotherapy, improving in the future the study and optimization of cancer therapy. We sought to identify a multifunctional imaging protocol for longitudinally studying the impact of T cell therapy and elucidating optoacoustic signatures that could reveal unknown aspects of immunotherapy through the entire tumour mass. Optoacoustic imaging resolves optical absorption contrast in high resolution, therefore allowing for a detailed visualization of tumour oxygenation^19^, vasculature and other optical alterations indicative of morphological and physiological information revealed at resolutions of a few tens (mesoscopy) to hundreds (macroscopy) of microns. We explored this information to resolve activity within the tumour mass and therefore spatially assessing therapeutic heterogeneity^20^. This feature can be potentially employed for monitoring tumour pathophysiological parameters occurring during immunotherapy progression.

For understanding immunotherapy at different scales, we combined the imaging characteristics of Multi-Spectral Optoacoustic Tomography (MSOT)^21^, appropriate for whole body animal imaging with 200–300 micron resolution and Raster Scan Optoacoustic Mesoscopy (RSOM), revealing optical features such as vasculature with resolutions better than 10 microns through 3–4 mm of tissue^22^. Based on the unique optoacoustic performance, we observed over time therapeutic effects manifested as optical changes across the tumour mass using MSOT and obtained a detailed view of vessel regression during tumour elimination by CTLs using RSOM. Multispectral excitation was further employed for resolving pathophysiological changes within the tumours through the differentiation of tumour intrinsic molecules. In particular, optoacoustic imaging showed capability of monitoring tumour pathophysiology by resolving *in vivo* the process of T cell induced tumour rejection through the detection of intrinsic tissue chromophores accumulated in necrotic tumour areas. In addition, we analysed the biodistribution of T cells by optimizing T cell labeling through the combination of two near-infrared fluorescent dyes with overlapping absorption spectra but different binding capacities and performed *ex vivo* episcopical fluorescence imaging after the last *in vivo* imaging time point. Our multi-spectral and multi-scale analysis revealed that non-invasive label-free imaging *in vivo* using MSOT and RSOM can offer high specificity in assessing therapeutic efficiency that can complement studies of T cell presence in the tumour and, importantly, directly observe the function of the cells, rather than solely their biodistribution. In this role, multi-scale optoacoustics are shown well suited for understanding and optimizing immunotherapy protocols. The addition of episcopic fluorescence imaging of the T cell biodistribution provides complementary information on T cell presence, in addition to therapeutic effects resolved by optoacoustics. We further introduce and explore a novel imaging workflow to analyse cellular immunotherapy of established solid tumours *in vivo* with optoacoustic and episcopic fluorescence imaging and demonstrate its benefit by retrieving information about tumour size, tumour vasculature and tumour pathophysiology in high resolution.

## Results

### Multiscale anatomical imaging of tumours during immunotherapy

To assess whether optoacoustic tomography could reveal anatomical information related to cancer immunotherapy, we imaged two groups of B6-Albino (B6-A) mice (n = 12) with s.c. tumours in the mammary gland overexpressing the chicken ovalbumin protein. One group (n = 6) was treated with tumour-antigen-specific T cells derived from OT-1 mice. This group, hereby referred to as the therapy group, was used for monitoring anti-tumour response. The second group (n = 6) received T cells from wildtype B6 mice, hereby referred to as the control group.

Longitudinal *in vivo* MSOT and RSOM^23^ was administered at indicated time points. Immediately after the last imaging time-point, mice were sacrificed and further submitted to histological analysis.

**Figure 1:**
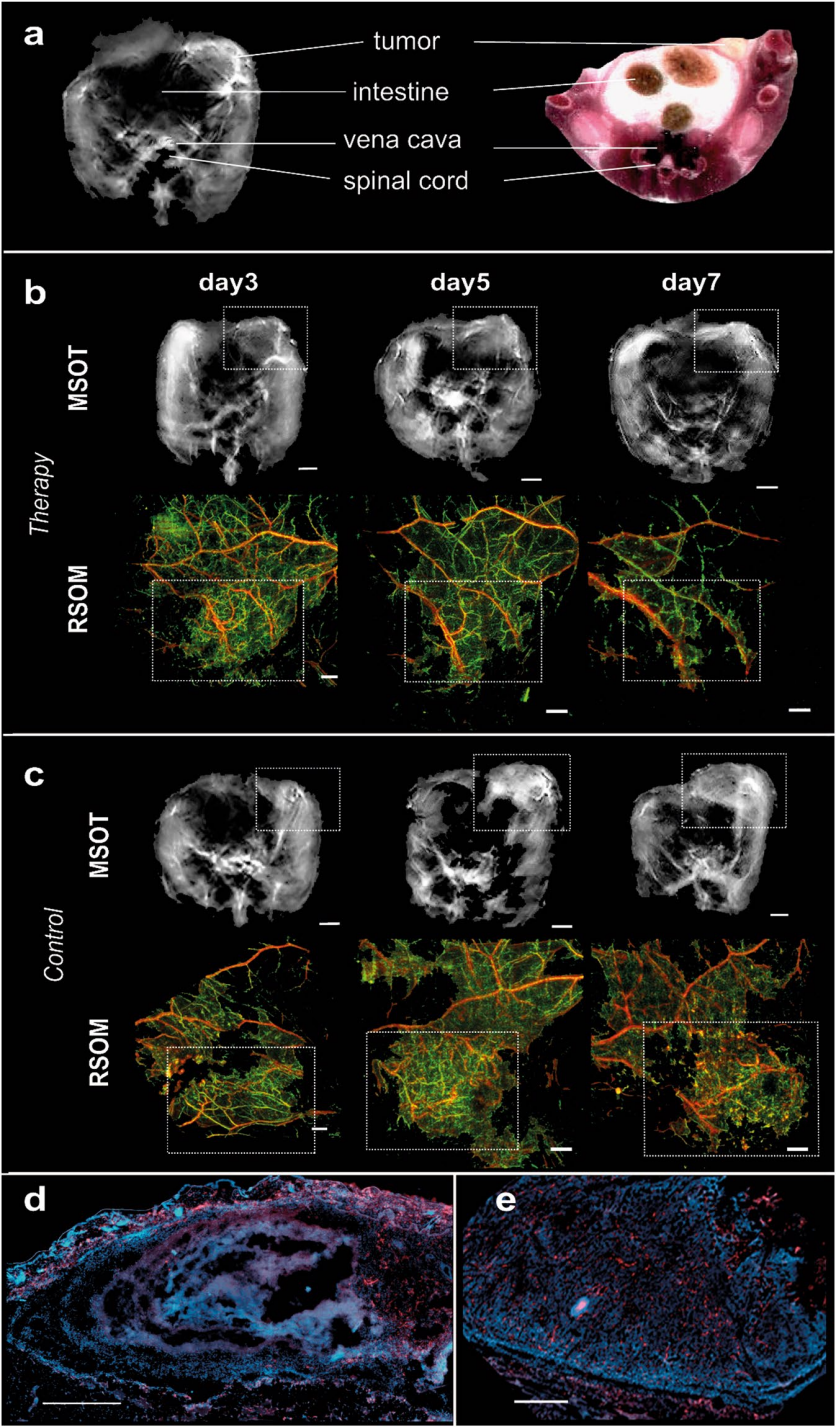
Longitudinal multi-scale imaging of anatomical tumour characteristics during immunotherapy. (**a**) Anatomical whole-body optoacoustic tomography (left) and corresponding cryoslice image (right) with indication of prominent tissue markers. MSOT (**b,c** upper row) and RSOM images (**b,c** lower row) of one representative animal corresponding to the therapy (**b**) and the control group (**c**). The tumour area is marked by a rectangle. In the RSOM image large vessels are colour-coded in red, small vessels in green. Mice of both groups were imaged at day 3, day 5 and day 7 after T cell transfer. (**d**,**e**) Merged CD31 (red signal) and DAPI (blue signal) immunofluorescence of the tumours presented in (B) and (C). A lack of the vasculature in the core of the tumour due to cellular decline is visible in the therapy group (**d**). The vasculature in the control tumour is unaffected both in the core and the periphery (**e**). Scale bar (RSOM) = 1 mm, scale bar (MSOT) = 2.5 mm, scale bar (IF) = 0.5 mm.

Examination of MSOT images revealed in all animals the morphology of the entire tumour mass (Fig. 1a–c). The differential contrast of the tumour vs soft tissue due to the intrinsic contrast of vasculature allows for an approximate location of the tumour area. Tumours in mice of the therapy group responded to the transferred OT-1 T cells and demonstrated a decreasing size over a period of up to 9 days (Fig. 1b, upper row), whereby tumours of the control group kept growing and tumour mass increased (Fig. 1c, upper row). The images further revealed, in both groups, tumours at day 3 with highly heterogeneous intratumoural pattern, indicative of spatially varying vascularization. However, as treatment progressed, we observed a strong absorption from the tumours of the therapy group (Fig. 1b, day 7) associated with vascular collapse.

To better elucidate the vasculature alterations to treatment, we performed in-tandem imaging of RSOM and MSOT (n = 4) as RSOM offers about an order of magnitude better resolution than MSOT (30 vs 300 microns), but only reaches approximately 2 mm into the tumour and does not reveal the entire tumour mass as in the case of MSOT. Longitudinal *in vivo* RSOM images highlighted the destruction of the tumour vasculature after the transfer of tumour-specific T cells (Fig. 1b, lower row). While large and prominent vessels remained rather intact at all imaging time points (Fig. 1b, lower row, purple colour-coding), smaller vessels within the tumour appeared to collapse and almost disappeared after day 5 (Fig. 1b, lower row, green colour-coding). This effect became increasingly evident at later stages of immunotherapy as the tumour mass decreased. In contrast, the vasculature within tumours of the control group appeared dense and highly branched at all time points and illustrated the formation of new vessels during tumour growth (Fig. 1c, lower row).

The longitudinal *in vivo* optoacoustic data appeared in agreement with post mortem histological analysis of tumour vessels (Fig. 1d,e). In the case of the therapy group CD31 immunofluorescence of the tumour tissue (Fig. 1d, red signal) revealed a rather diffuse signal in the tumour core indicating the absence of intact vessels in this area. The vasculature in the skin and at the edges of the tumour however remained intact. In the case of the control group vessels were present both in the tumour periphery as well as in the tumour core (Fig. 1e). Additional histological investigations confirmed cellular decline within therapeutic responding tumours correlating to the signal-responding areas in MSOT (Fig. S1).

### Label-free imaging of tumour rejection through the detection of cellular decline

Aside to high-resolution anatomical imaging, MSOT allows for performing molecular imaging by separating the spectral signatures within tissues. For investigating pathophysiological changes of tumours undergoing therapy, we obtained multispectral optoacoustic images of all animals of the therapy and the control group (n = 12) at 21 different wavelengths ranging from 700 to 900 nm.

**Figure 2:**
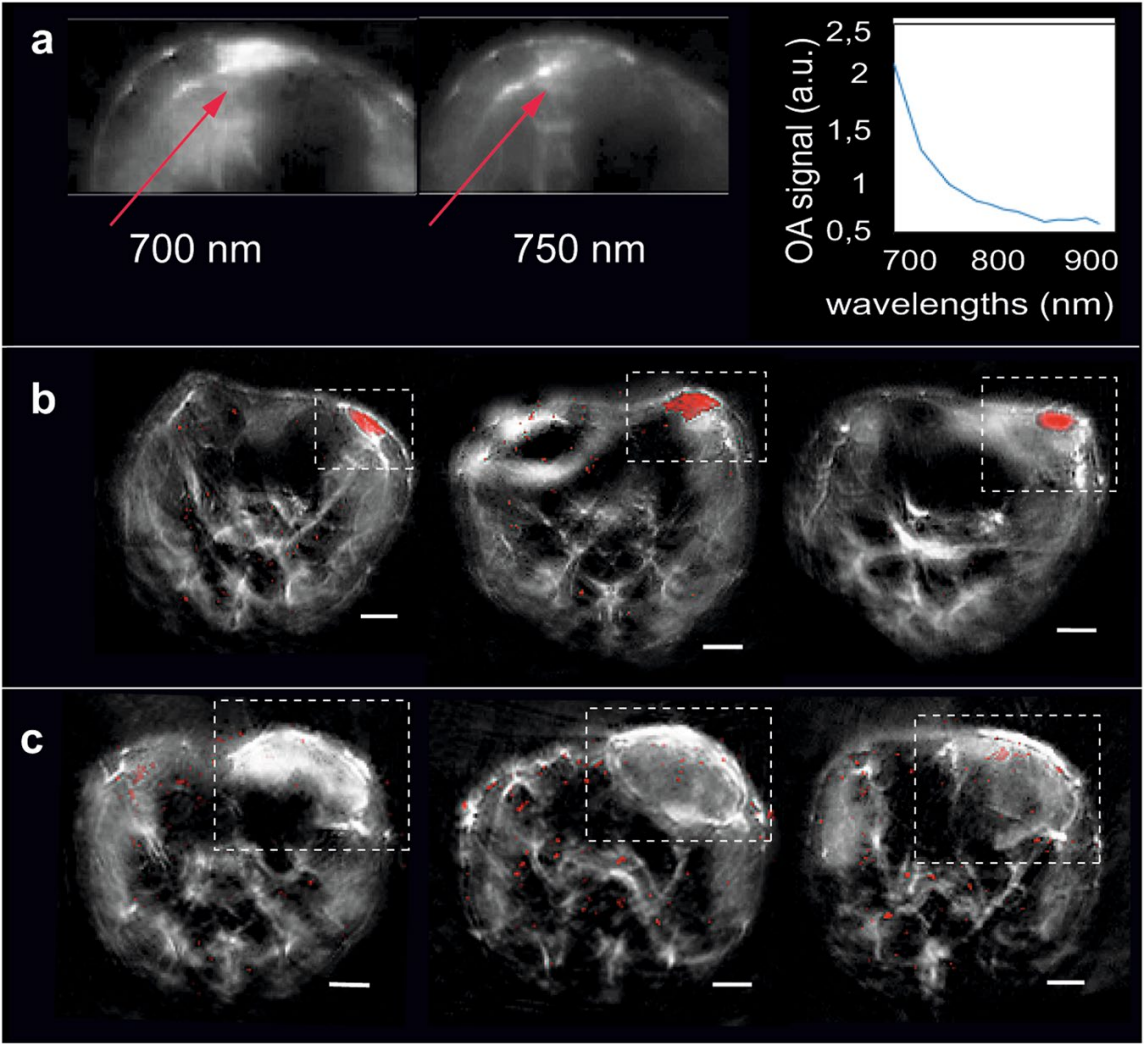
MSOT imaging of tumour behaviour during T cell therapy. (**a**) Optoacoustic images at different excitation wavelengths (700 nm, left and 750 nm, right) reveal a gradient signal in animals of the therapy group. The spectrum of the gradient signal is shown in the graph. (**b,c**) Spectral detection of the gradient signal within tumours of 3 representative animals of the therapy (**b**) and the control group (**c**) at the last imaging time-point (up to day 9 after T cell transfer). The gradient signal (red colour) is consistently detected in the tumours (labeled) of all animals of the therapy (**b**) but not in the case of the control group (**c**). Scale bar (MSOT) = 2.5 mm.

Inspection of MSOT images from all animals revealed spectral differences depending on the therapeutic outcome. Figure 2a demonstrates the tumour of one representative animal of the therapy group with tumour parts showing high absorption at low wavelengths (700 nm) that rapidly decreased at higher wavelengths (750–900 nm). Spectral analysis by means of AMF (Methods) was used for detecting this gradient absorption signal with the spectrum presented in Fig. 2a. Interestingly, the gradient signal was detected only in animals of the therapy group where tumours responded to the adoptively transferred tumour-specific T cells and large areas of cellular decline were detected. We present merged MSOT images (red colour-coding indicates the gradient signal) of three representative animals of the therapy (Fig. 2b) and the control group (Fig. 2c). As the signal does not bear resemblance to the spectra of oxygenated and deoxygenated hemoglobin, which are the main tissue absorbers, it is most likely that it correlates to degrading products (e.g. lipids or fatty acids) of declined tumour cells. Histological analysis of the tumours indeed identified apoptotic and necrotic areas in tumours undergoing immunotherapy whereas tumours of the control group remain vital after the transfer of tumour-unspecific T cells (Fig. S1).

**Figure 3:**
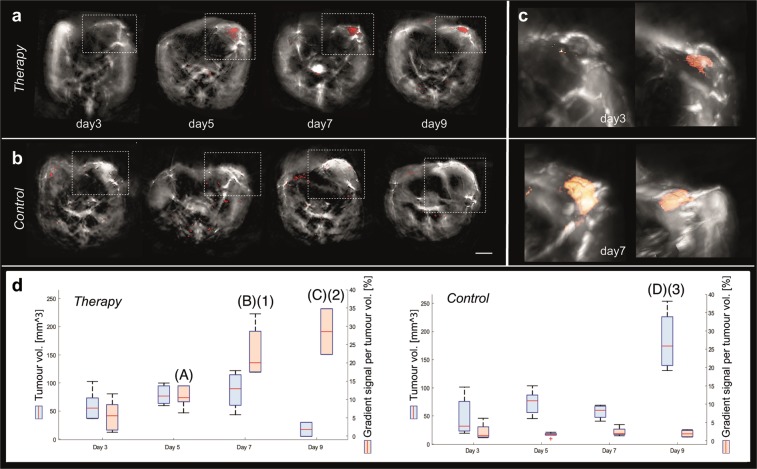
Longitudinal assessment of tumour rejection using MSOT. (**a,b**) Merged MSOT images of the gradient signal indicating cellular (red colour-coding) overlaid onto the anatomical optoacoustic images. One representative animal of the therapy (**a**) and one of the control group (**b**) is shown. Animals were imaged every second day starting at day 3 after T cell transfer. (**c**) 3D maximum intensity projection rendering of the merged MSOT images for the whole tumour volume of the animal shown in (**a**). Scale bar (MSOT) = 2.5 mm. (**d**) Tumour volumes (in mm^3^, blue colour-coding) from animals of the therapy (left image) and the control group (right image). The gradient signal per tumour volume (in %) is illustrated in red. Values are given as boxplots (mean; whiskers: min to max). Day 3 was used as baseline. Statistically significant differences of the gradient signal per tumour volume within the therapy are depicted above the relative day (**A,B,C**). The statistically significant difference of the tumour volume within the control group is marked for day 9 (**D**). Statistically significant differences of the gradient signal per tumour volume between the two groups (therapy and control) are depicted in the therapy group for day 7 and day 9 (1,2) and for the tumour volume in the control group at day 9 (3). *p* ≤ 0.05.

Label-free MSOT imaging offers the ability to longitudinally monitor the process of tumour rejection at different stages of immunotherapy and assess the time point when tumours start to undergo physiological changes without the need of contrast agent. In all animals of the therapy group, the gradient signal starts to be weakly detectable at day 3 after T cell transfer (one representative animal shown in Fig. 3a). From day 5 on, the gradient signal became apparent in parts of the tumour of all mice belonging to the therapy group, indicating cell death due to cytotoxic T cell (CTL) activity (Fig. 3a). The gradient signal became increasingly evident the further therapy progressed (Fig. 3a), while it was not evident in the tumours of the control group at any imaging time point (one representative animal shown in Fig. 3b). The detection of the gradient signal only at distinct sub-regions of the tumour reveals the heterogenous process of tumour rejection in parts due to the CTLs activity.

As MSOT imaging was done in three dimensions, reconstruction of 3D images was possible and facilitated the answer to spatial distribution questions (Fig. 3c). Accordingly, information about depth is possible next to the precise location. Due to the spatial information we obtained with MSOT we were able to analyse the tumour volume until day 9 after T cell transfer and determined the percentage of gradient signal within the tumour (Fig. 3d). In the therapy group the tumour volume remained nearly constant until day 7 and then decreased until day 9 after T cell transfer. In contrast the percentage of gradient signal per tumour volume increased over the entire trial period. Using day 3 as baseline value we have compared the tumour volume and the signal intensity per tumour volume within each experimental group (therapy and control) (Fig. 3d, letters). Within the therapy group we obtained significant differences (*p* ≤ 0.05) in the percentage of gradient signal per tumour volume at day 5, day 7 and day 9 (Fig. 3d, A/B/C). Within the control group there was no significant differences for the gradient signal per tumour volume found. Concerning the tumour volume we found a significant difference at day 9 (Fig. 3d,D) again using day 3 as baseline value. Next, we examined potential inter-group differences between the two data groups. The percentage of gradient signal per tumour volume of the therapy and the control group was significant different at day 7 and day 9 (Fig. 3d, 1/2). The tumour volume showed only at day 9 a significant difference between the two groups (Fig. 3d, 3).

Hence, detection and examination of the gradient signal using MSOT technology, we were able to determine tumour rejection in a label-free manner.

### Combined imaging of labeled T cells and tumour pathophysiology

To elucidate the tumour pathophysiological results obtained label-free by MSOT, in conjunction with the T cell biodistribution, we performed combined *in vivo* MSOT and *ex vivo* episcopic fluorescence optical imaging of mice treated with fluorescently labeled T cells (n = 10). T cells were labeled using a combination of a cell membrane staining near-infrared (NIR) dye (DiR) and a preferrentially cytoplasm staining NIR dye (VivoTag-S 750). In this manner, we were able to optimize T cell signal intensity without compromising cell viability or specificity (Fig. S2). Furthermore, we performed an *ex vivo* study to detect the sensitivity of MSOT for double labeled T cells. By doing so, we achieved a sensitivity limit of 20.000 T cells in a volume of 50 µl (Fig. S3).

**Figure 4:**
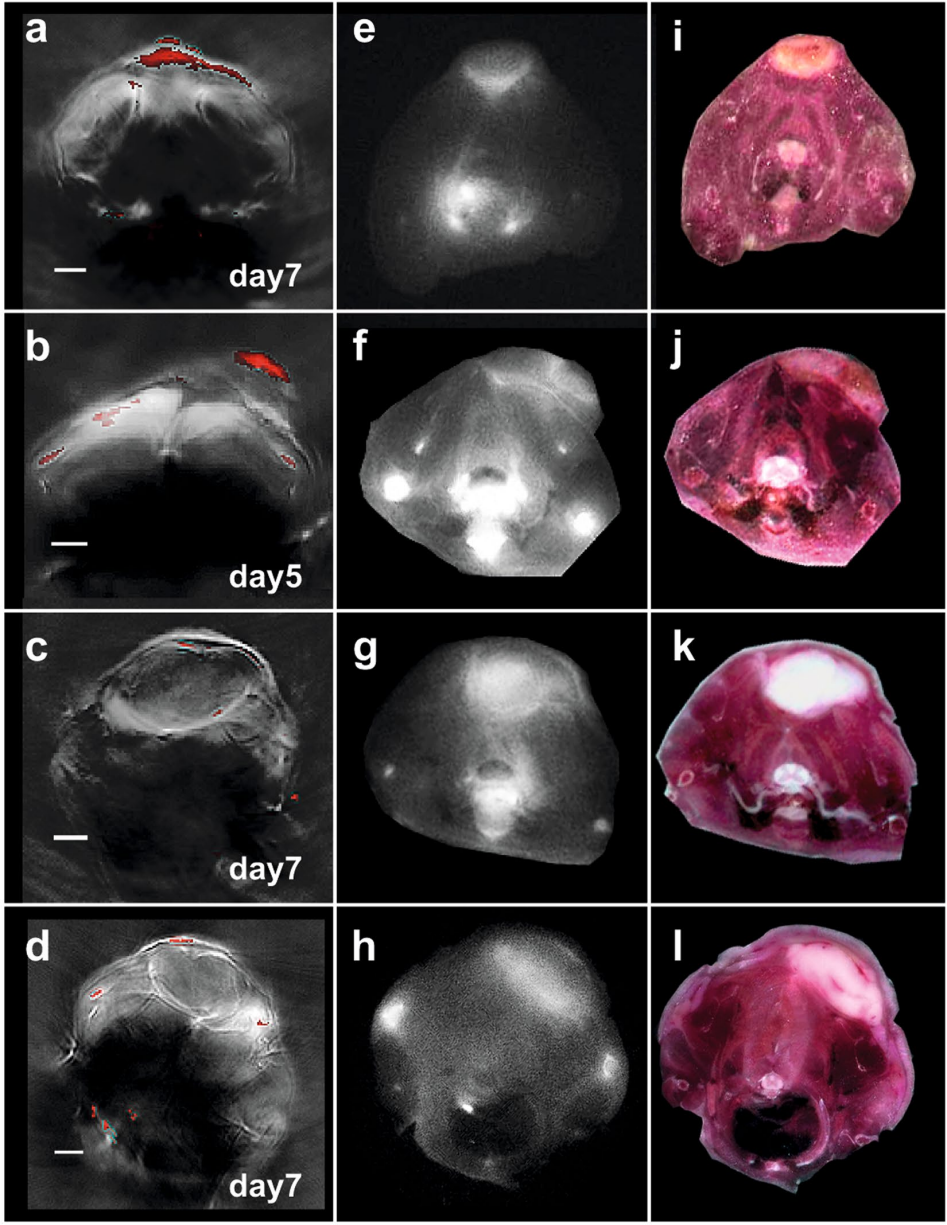
Multimodal analysis of T cell-based immunotherapy. (**a–d**) MSOT images of animals from the therapy (**a,b**) and control group (**c, d**) at indicated time points. Red colour-coding corresponds to the gradient signal. (**e-h**) Episcopic fluorescence imaging of the mice presented in (**a–d**). Accumulation of double labeled T cells was found in tumours of both the therapy (**e,f**) and the control group (**g,h**). Within the therapy group, T cells are concentrated around the gradient signal. In the control group, T cells can also be found at the tumour edge and within the tumours. (**i,j**) Cryo RGB image correlating to the episcopic fluorescence images. In tumours of the therapy group (**i,j**), cellular decline is visible in central parts of the tumour. Tumours of control animals (**k,l**) do not present large areas of cell death. Scale bar (MSOT) = 2.5 mm.

Double labeled T cells were transferred into mice of the therapy (n = 6) and control group (n = 4) and MSOT images prepared. After MSOT imaging at predefined time-points, animals were sacrificed and analysed using episcopic fluorescence imaging. Aim of this multi-modal imaging approach was to analyse the spatial T cell distribution in the tumour and their biodistribution at different time points after transfer with high resolution. Figure 4a–d presents merged MSOT images (red colour-coding indicates the gradient signal) of two representative mice corresponding to the therapy (Fig. 4a,b) or the control group (Fig. 4c,d). As observed before, the gradient signal was found in mice of the therapy group predominantly in a central position within the tumour (Fig. 4a,b). Interestingly, the gradient signal appeared to be adjacent to areas with increased fluorescence intensity which points to the presence of transferred labeled T cells (Fig. 4e,f). Analysis of the corresponding cryoslice images (Fig. 4i,j) confirmed that the area in which the gradient signal is located presented cell death whereas the T cells had settled in or at the edge of vital tumour tissue. In addition, H&E staining confirmed that the tumour areas in which the gradient signal is detected feature cellular decline (data not shown).

Tumour-unspecific B6 T cells though also home to the tumour mass and were detectable even until day 7 after transfer (Fig. 4g,h). However, no gradient signal was found in any of these tumours (Fig. 4c,d) and neither cryoslice images (Fig. 4k,l) nor H&E staining (data upon request) did present large areas of cellular decline. Nevertheless, the tumour-unspecific T cells seem to surround the tumour mass, but also infiltrated the tumour even several days after transfer.

Concerning the biodistribution, T cell homing to the bone marrow was visible in several mice of both, the control and the therapy group (Fig. 4f,g). In addition, MSOT detected high accumulations of T cells in the lymph nodes of all mice analysed (Fig. S4).

## Discussion

*In vivo* imaging is critical to understand the function and interaction of immune cells in their native environment, and helps to optimize cancer immunotherapy strategies. Despite progress with clinical trials^2,24–26^, successful monitoring of immunotherapies remains a major challenge and an important goal for better understanding and optimizing immunotherapies. Methods like biopsies or *ex vivo* analysis of cell functions give a time-restricted picture of cell trafficking, homing and distribution prerequisites for a successful therapy that can be studied by *in vivo* imaging. Optical imaging is well suited for studying the longitudinal course for cell distribution but is limited by the low penetration depth of microscopy or the two-dimensional, low resolution views of bioluminescence or fluorescence imaging.

In this work, we introduce statistical sup-pixel detection MSOT as a highly potent alternative optical modality with the ability to offer three-dimensional high-resolution views of tumours undergoing immunotherapy *in vivo*, and reveal underlying morphological and physiological tumour features. In our view, this is an imaging capacity allowing observations of cell biology much deeper (several cm) than what was allowed today by established optical imaging modalities (beneath 1 mm)^27^.

We employed a combination of small-animal MSOT and RSOM in an established mouse model for adoptive cell therapy^28^ for obtaining high resolution information on tumour vasculature and correlated the process of tumour regression with the process of vascular collapse due to the action of CTLs. This ability of optoacoustic imaging systems cannot be offered by intra-vital microscopy where the imaging window and the penetration depth limit the ability to obtain a holistic tumour view. In addition, RSOM is superior over fluorescent microscopy as it allows label-free imaging of vessels. Changes in the tumour vasculature are of special interest within the establishment of effective cellular immunotherapies as tumour vessel normalization leads to an increased tumour response to transferred T cells^29^ and CTLs have been reported to efficiently destroy tumour vessels in established solid tumours^30^.

The mechanisms by which T cells kill tumour cells or modify the tumour microenvironment is still not fully resolved. Amongst others, tumour necrosis factor-α (TNF-α) and IFN-γ are two major effector molecules in tumour destruction by CTL both targeting the tumour vasculature^31,32^. Besides, IFN-γ can act directly on tumour cells, but also remodels the microenvironment^33^. Kammertoens *et al*. showed the effect of IFN-γ on endothelial cells and the necessity of this interaction on the eradication of large established solid tumours^34^. Townsend *et al*.^35^ demonstrated that T cell infiltration and tumour elimination are dependent upon the degree of tumour vasculature and the corresponding oxygenation^35^. In addition to many other criteria, this shows the attractivity and importance of imaging tumour vasculature in preclinical and clinical studies. Using RSOM as label-free, 3D imaging system for visualizing the tumour vasculature will allow fast and non-invasive longitudinal studies *in vivo*.

A key advantageous MSOT feature, with significant future potential, is the ability to measure a number of underlying morphological, physiological or biochemical tumour features like the process of tumour regression in a label-free manner through the detection of an intrinsic absorption signal related to cell death. The application of multiple wavelengths for exciting tissues and molecules and analysing these different spectra one by one directed us to the intrinsic gradient signal occurring in therapeutic responding tumours only. The gradient signal we identified (rapid decrease from 700 nm to 900 nm) was found to correlate well with cellular decline within the tumour tissue as assessed through histological analysis. Our observations are in accordance with previous reports stating that molecular and physiological changes within tumours undergoing T cell-based immunotherapy typically starts around day 3 after T cell transfer^7,12^. To our best knowledge, it is the first time that such an ability has been reported.

To complete multi-imaging analysis of adoptively transferred T cells, we analysed T cell biodistribution using *ex vivo* episcopic fluorescence imaging. To achieve the most optimal signal we improved the cell labeling technique through the incorporation of two near infrared fluorescent dyes with identical spectra. We took advantage of an amine-reactive, predominantely cytoplasmic staining dye (VivoTag-S 750) and a membrane-intercalating (DiR) dye and optimized cell labeling in a way that cell viability and functionality was not compromised. As T cells do not exhibit large cytoplasmic regions (compared to microphages) and have a diameter of ~10 µm in the activated state, labeling is challenging. Alternative labeling strategies, for example using gold nanorods, may impair important cellular functions by the uptake of sufficient amounts of gold. Alternative labeling strategies could consider reporter gene technologies, for example using the tyrosinase gene^36^, which could lead to improving the cell detection sensitivity. Biodistribution of transferred cells is of high interest as off-site targets were reported to cause severe adverse events^37^. Furthermore, studying the biodistribution of different T cell subsets (e.g. naive vs effector memory vs central memory T cell) *in vivo* is aim of several studies to identify the most optimal subset for immunotherapies^38^.

Most imaging efforts so far have focused on imaging the distribution of labeled T cells during the course of immunotherapy only. However, there is a need for imaging methods that can reveal *in vivo* tumour anatomical and pathophysiological features related to therapy both for assessing therapy outcome and for better understand the mechanisms of tumour cell killing. We found that multi-functional multi-scale and multi-spectral optoacoustic imaging can complement current imaging efforts with the ability to offer three-dimensional high-resolution views of tumour anatomy and pathophysiology.

## Materials and Methods

### Animal procedures

C57Bl/6-Albino (B6-A), C57Bl/6 (B6) and C57BL/6-Tg(TcraTcrb)1100Mjb/J (OT-1) mice transgenic for a Vα2, Vβ5 T cell receptor reacting with an octamer peptide of ovalbumin (OVA_257–264/SIINFEKL_) in association with H-2K^b^ derived from approved experimental animal breedings (e.g. Taconic, Charles River Laboratories). For the experiments, 8–12-week-old female mice were used. All procedures involving animals were in conformity with national and institutional guidelines, approved from the animal ethics committee of the local authority (Regierung von Oberbayern, Munich, Germany) and supervised by the Institutional’s Animal Care and Use Committees (AVM, Helmholtz Zentrum München, Neuherberg, Germany and ZPF, Klinikum rechts der Isar, Munich, Germany). Animals were housed in standard animal laboratories (12 h light/dark cycle, 50–60% humidity) in individually ventilated cage systems (IVC Techniplast) under pathogen-free conditions with free access to water and standard laboratory chow *ad libitum*. In total, 24 B6-A mice were subcutaneously injected with 0.5 × 10^6^ EG7 cells in according to standard protocols. Each control or experimental group was composed of 6 animals which were chosen randomly at the beginning of the experiment. Sample size was chosen based on power analysis. Tumour sizes were measured using a caliper (FST) and tumour volume calculated using the formula *tumour vol [mm*^3^] = *0.5* × *(length* × *width*^2^*)*. At the time of T cell transfer (tumour size 10–40 mm^3^), 1 × 10^6^ unlabeled or 1 × 10^7^ double labeled OT-1 and B6 T cells were systemically injected. For *in vivo* studies, animals were deeply anesthetized using 1.8% Isoflurane with medical O_2_ (0.8 l/min) and imaged at indicated time points (n = 12) or longitudinally at intervals of 36 hours with a maximum of 5 scans per animal (n = 12). After the final time-point of imaging (end-point of the experiment) animals were sacrificed under anesthetic using standard procedures and finally submitted to episcopic imaging using a fluorescence cryoslicing set up^39^.

### Tumour cell lines

Chicken ovalbumin expressing EG7 cells (ATCC, CRL-2113^TM^) and its parental cell line EL4 (ATCC TIB-39^TM^) were maintained in RPMI 1640 medium (Gibco) containing 10% FBS, 1% Na-Pyruvat, 2 mM Glutamine, 50 µM 2-ME, 100 IU/ml penicilin/streptamycin (T cell medium). In addition, EG7 culture medium contained 0.4 mg/ml g418 (Life Technologies).

### T cell isolation, expansion and labeling

Single T cell suspensions derived from erythrocyte-depleted spleen cell suspensions of OT-1 and B6 mice (155 mM NH_4_Cl, 10 mM KHCO_3_, 0,1 mM EDTA) by activation of 2 × 10^6^ cells/ml splenocytes in T cell medium for 48 hours. OT-1 T cell suspensions were supplemented with 2 µg/ml 50 U/ml rm IL-2 (R&D) and 0.1 µg/ml CD28 (R&D), B6 T cell were activated in CD3 coated cell culture flasks (1 µg/ml, R&D) supplemented with 50 U/ml rmIL-2 and 0.1 µg/ml CD28. Next, cells were expanded at a density of 1 × 10^6^ cells/ml in T cell medium supplemented with 40 ng/ml rmIL-15 (R&D) for additional 3 days. On day 5 after isolation, cells were labeled with 10 µM Xenolight DiR (Perkin Elmer) in 1x PBS at a concentration of 1 × 10^6^ cells/ml for 15 minutes at 37 °C, with 24 µM VivoTag-S 750 (Perkin Elmer) in T cell medium at a concentration of 4 × 10^6^ cells/ml for 30 minutes at 37 °C or double labeled with both dyes. After washing twice with 1x PBS, labeled T cells were incubated for 2 hours in T cell medium with 40 ng/ml IL-15 to recover. Subsequently, labeled cells were transferred into animals or further analysed *in vitro*. Labeling conditions for VivoTag-750 were analysed using full medium and serum-free medium and the same conditions as described earlier.

### T cell analysis

Effectivity of labeling was analysed by fluorescence microscopy (Axio Imager Z1, Carl Zeiss) and flow cytometry (Cyan ADP, Beckman Coulter) using appropriate software (AxioVision, Carl Zeiss; FlowJo, Tree Star Inc.). Flow cytometry measurements were done with ~0.5 × 10^6^ unlabeled, single or double labeled cells fixed in 4% PFA and stored in 1xPBS/0.2% FBS/0.1% NaN_3_. Cell viability was examined by the trypan-blue exclusion test (Sigma-Aldrich) and XTT [2,3-Bis-(2-methoxy-4-nitro-5-sulfophenyl)-2H-tetrazolium-5-carboxanilid-salt] assay (Abnova) according to standard procedures. XTT assay was performed in triplets with 20.000 cells per well.

### ELISA

For the detection of the IFN-γ production by OT-1 T cells, 4 × 10^4^ T cells/well were cocultured with 2 × 10^4^ target cells (EL4, EG7) in 96-well round- bottom plates at 37 °C. After 24 hours, supernatants were analysed for IFN-γ content by enzyme-linked immunosorbent assay (ELISA; BD Pharmingen International) according to manufacturer’s protocol.

### *In vitro* T cell absorbance characterization

To assess the optoacoustic signal produced by NIR-labeled T cells, cells were first imaged *in vitro* by utilizing 2 cm –diameter cylindrical phantoms made of 1.3% Agarose (Sigma-Aldrich) and 1.2% intralipid emulsion (Sigma-Aldrich) leading to an optically diffusive medium. Intralipid was employed to impart scattering properties in the phantom, similar to those of tissue, and achieve a diffusive illumination, homogeneously illuminating object placed in the middle of the phantom. By not adding an absorber, we opted for a low absorption background to explicitly study the optoacoustic signal produced by the labeled cells decomposed from attenuation and phantom geometry effects. The labeled T cells in a mixture of 1x PBS and low melting point agarose (Sigma-Aldrich) at a concentration of 10^6^ cells/300 μl were enclosed within a 3 mm diameter plastic cylindrical tube implanted into the agar cylinder. The effective volume contributing to the optoacoustic signal was computed to be 5.6 μl, based on the diameter of the cylindrical tube and the elevation resolution of the MSOT scanner (~800 μm). The number of cells contributing to the optoacoustic signal was calculated from the effective volume and the cell concentration as 18.700 cells. Imaging was performed at the wavelength of 760 nm where the labeled cells exhibit and absorption peak. Next to the cells, we imaged a solution of water and black india ink (Higgins) with measured absorbance of 0.5 cm^−1^ at 760 nm. By comparing the signal contribution of the labeled cells to the India ink solution we were able to assess the absorbance of the cells.

### *In vitro* sensitivity study

We used a CD-1 nude mouse for post mortem s.c. injection of 100.000, 75.000, 50.000, 30.000, 15.000 and 5.000 labeled T cells in 50 µl phenol-red free, growth factor reduced matrigel (Corning). In the following, the mouse was imaged using episcopic imaging and MSOT at wavelengths 700 nm, 730 nm, 760 nm, 790 nm, 805 nm, 820 nm, 835 nm, 850 nm and 865 nm. The data were spectrally analysed to identify the lowest detectable amount (concentration) of cells using MSOT. Spectral analysis was performed by means of AMF (Adoptive Matched Filters)^40^.

### RSOM imaging

RSOM imaging was performed using an in-house-built raster-scan optoacoustic mesoscope^41^. Optical excitation is achieved by using a fast ns-laser operating at 2 kHz and 532 nm (Wedge HB 532, Bright Solutions). For the acoustic detection, we used a spherically focused high frequency detector at 50 MHz with an f-number of 1.3 (Precision Acoustics), the signal is further amplified by a low noise 63 dB amplifier (AU-1291, Miteq Inc.), and digitized at 500 MSps (Gage-Applied). The acoustic bandwidth is approximately 80%. For generating a three-dimensional image, we scan the detector in the xy-plane, and subsequently applying three-dimensional beamforming. Finally, before reconstruction, we filter the signals into two sub-bands, low frequencies and high frequencies, which we reconstruct separately, and then overlay using different colours for better visualization of the microvasculature.

### MSOT imaging and spectral unmixing

Optoacoustic imaging was performed using a commercially available “In Vision” MSOT scanner (iThera-Medical GmbH). Optical excitation is achieved by using an OPO tunable laser. Ultrasound detection is achieved using a cylindrically focused 256-element transducer array with 5 MHz central frequency (−6 dB of >50%), covering an angle of 270 degrees around the sample. The radius of the cylindrical array is 4.14 cm. The system acquires cross-sectional (transverse) images through the animal with an effective field of view of 2.5 cm × 2.5 cm, adequate for imaging through the whole body. By translating the animal through the focal plane of the ultrasound array and acquiring several cross-sectional slices allows for reconstructing three dimensional images after 3D rendering. Image acquisition speed for a single slice and wavelength is defined by the repetition rate of the laser (10 Hz). Multispectral imaging was performed by utilizing 9 excitation wavelengths, namely 700 nm, 730 nm, 760 nm, 790 nm, 805 nm, 820 nm, 835 nm, 850 nm and 865 nm.

### Quantification of tumour volume and gradient signal

For the quantitative assessment of tumour growth and gradient signal distribution, the tumours were segmented manually from the optoacoustic images. The voxel size was estimated from the image resolution and scanning step in z direction. Tumour volume was estimated by multiplying the amounts of voxels in the segmentation masks by the voxel size. The presence of the gradient signal was quantitatively assessed by dividing the volume within the given tumour where the gradient signal was detected by the total volume of the tumour. Mean values and standard deviation were calculated for all individual parameters.

### Statistical analysis

Data was analysed using MATLAB (VersionR2017b, TheMathWorks, Natick, USA). Differences in tumour size and MSOT signal intensity of the gradient signal per tumour volume within the control and therapy group and between the groups, were calculated by the Student’s unpaired two-sample *t*-test. For intra-group calculations, data from each day was compared to the data from day 3 as baseline. For inter-group calculations, data was normalized by deviding the data by the mean of day 3 (per group) to get changes relative to the baseline and comparing the results to the normalized baseline measurements (day 3) of the other group. *p* values ≤ 0.05 were considered to be significant. Rejection of the null hypothesis at the 5% significance level was indicated above the corresponding data point.

### Episcopic fluorescence imaging, cryoslicing and histology

After the last MSOT imaging time point, animals were euthanized and frozen at −80 °C. Before slicing, the body region of interest was selected and embedded in black optimal cutting temperature compound (OCT, TissueTek). The slicing was performed using a cryotome (CM 1950, Leica Microsystems GmbH) as described in^42^. For episcopic fluorescence imaging, a custom made system consisting of a 750 nm-300 mW laser (BWTEK Inc.) and cold white light source (KL2500, Schott), 2-filter-wheels for excitation (FW102C, Thorlabs) and emission (10-pos. custom made, Prior Scientific) hosting reflection (RGB) and fluorescence (exc: 740/40/emm.: 780LP) filter sets (Chroma), a photographic lens with varying zooming and focus, a near-infrared electron multiplying charged coupled device (EMCCD Luca R, Andor) and a Labview (National Instruments Cooperation) based automated acquisition software was used. The exposure time was automatically selected to ensure optimal use of the dynamic range of the EMCCD sensor and the images were normalized in the post-processing to ensure comparable results. Representative thin-slices of 10 μm thickness were extracted on histology glasses for further histopathological analysis and stored at −20 °C. After pre-fixation in 4% paraformaldehyde (PFA) (Santa Cruz Biotechnology), hematoxylin and eosin staining was performed according to the manufacturer protocol (Morphisto). Sections were immunohistochemically stained for CD31 (Histonova), Caspase 3 (Merck) and Cytochrome C (CellSignaling) and visualized using Alexa Flour 594 (Life Technologies). Prolong Gold (LifeTechnologies) containing DAPI was used for tissue embedding and visualization of the cell nuclei. All slices were imaged using an Axio Imager M2 microscope (Carl Zeiss) equipped with the Zeiss Zen software (Carl Zeiss).

## Supplementary information

Supplementary Information.

## Data Availability

The datasets generated during and/or analysed during the current study are available from the corresponding author on reasonable request.

## Code availability

MSOT image reconstruction was performed per slice and excitation wavelength using a model-based inversion algorithm^43^. The T cell biodistribution was spectrally separated from the absorbing tissue background using the adaptive matched filter (AMF)^40^, the application of which in the context of multispectral optoacoustic tomography is explained in ref. ^44^.
