# Supplementary material for: Longitudinal imaging of T cell-based immunotherapy with multi-spectral, multi-scale optoacoustic tomography
Source: Sci Rep. 2020 Mar 17;10:4903. doi: 10.1038/s41598-020-61191-z (PMC7078227; doi:10.1038/s41598-020-61191-z)
Supplement: Supplementary file 1 — Supplementary Information. [file 41598_2020_61191_MOESM1_ESM.pdf]

Supplementary Information:

Longitudinal imaging of T cell-based immunotherapy with multi-spectral, multi-scale optoacoustic tomography

Melanie A. Kimm[1#], Stratis Tzoumas[2,3#], Sarah Glasl[2,3], Murad Omar[2,3], Panagiotis Symvoulidis[2,3], Ivan Olefir[2,3], Ernst J. Rummeny[1], Reinhard Meier[1], Vasilis Ntziachristos[2,3*]

[1] Department of Diagnostic and Interventional Radiology, School of Medicine & Klinikum rechts der Isar, Technical University of Munich, Munich, Germany

[2] Chair for Biological Imaging, School of Medicine & Klinikum rechts der Isar, Technical University of Munich, Munich, Germany

[3] Institute of Biological and Medical Imaging, Helmholtz Zentrum München, Neuherberg, Germany

# These authors contributed equally to this work.

* Corresponding author

Correspondance to:
Vasilis Ntziachristos,
Chair for Biological Imaging,
School of Medicine,
Technical University of Munich,
Ismaningerstr. 22, 81675 Munich, Germany
phone ++49-89-4140 7211
fax ++49-89-41406748
v.ntziachristos@tum.de

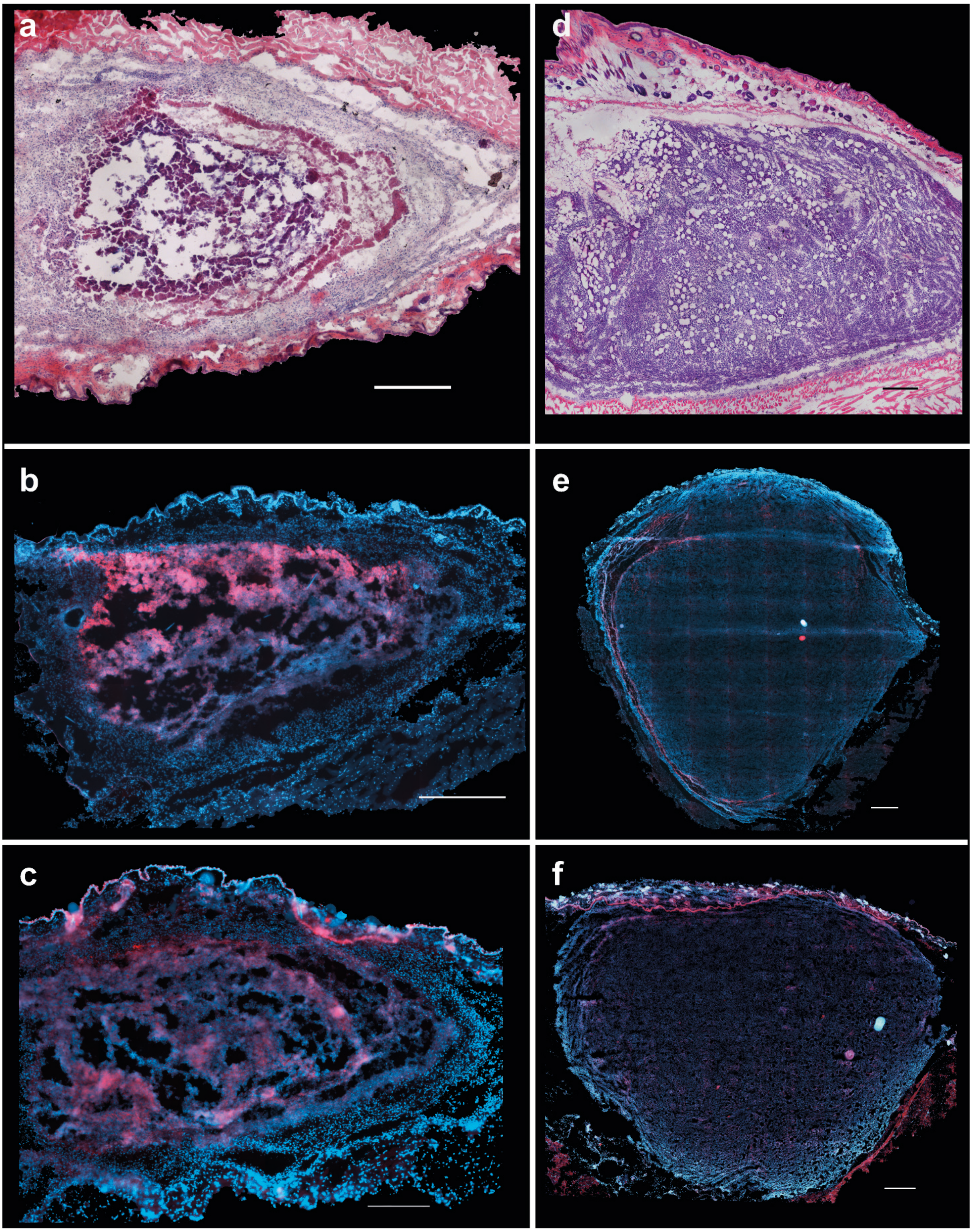

Figure S1. Histological analysis. One representative tumour of the therapy (**a-c**) and one of the control group (**d-f**) is shown. H&E staining (**a, b**) highlights necrotic and apoptotic areas within the tumour core of the therapy group (**a**) whereas the one of the control group presents virtal parts (**b**). Caspase 3 (**b, e**) and Cytochrome C (**c, f**) immunofluorescence (red) reveals apoptotic events only in the tumour of the therapy group (**b, c**). Nuclei counterstaining with DAPI (blue). Scale bar (**a, b, c**) = 200 µm, scale bar (**d, e, f**) = 500 µm.

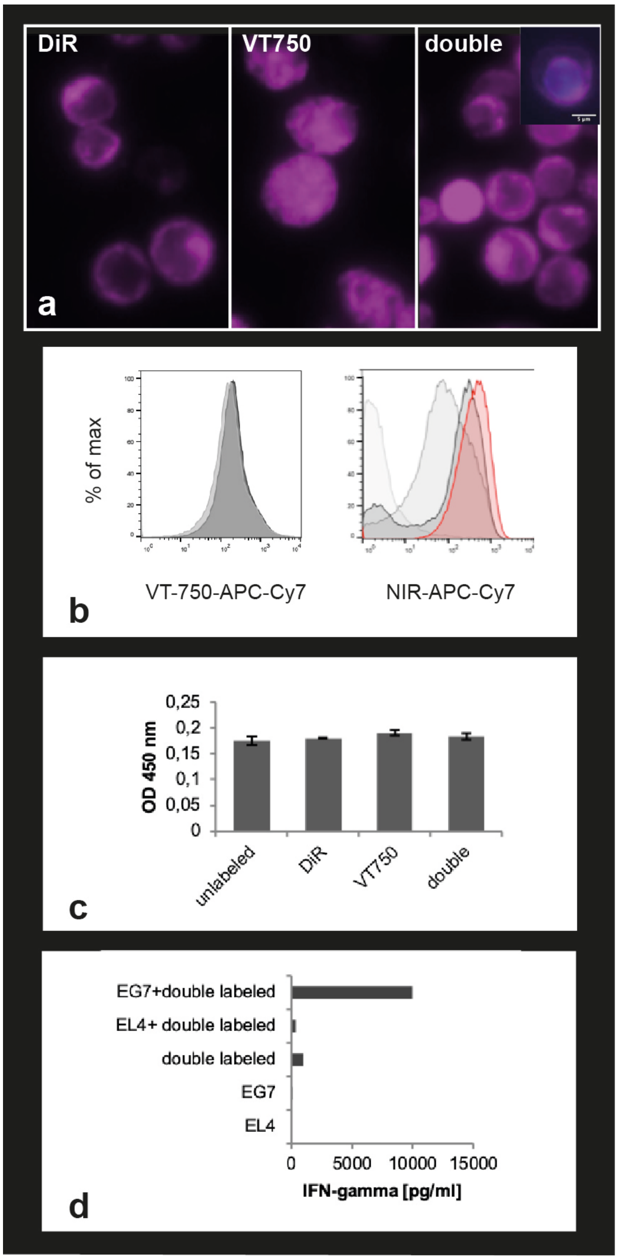

Figure S2. T cell viability and functionality following cell labeling. (**a**) Fluorescence microscopy images of DiR (left), VT-750 (middle) and double labeled OT-1 T cells (magenta). Nuclear counterstaining with DAPI (blue). Scale bar 5 µm. (**b**) Flow cytometry analysis of OT-1 T cells labeled with VT-750 in full medium (left image, light grey) or serum-free medium (left image, dark grey). Flow cytometry analysis of unlabeled, single and double labeled cells (right). (**c**) XTT cytotoxicity assay after cell labeling. (**d**) IFN-γ ELISA with double labeled OT-1 T cells. OVA-expressing EG7cells were used as positive control and parental EL4 cells as negative control. Cells were co-cultured for 24 hours at responder: stimulator ratio 2:1.

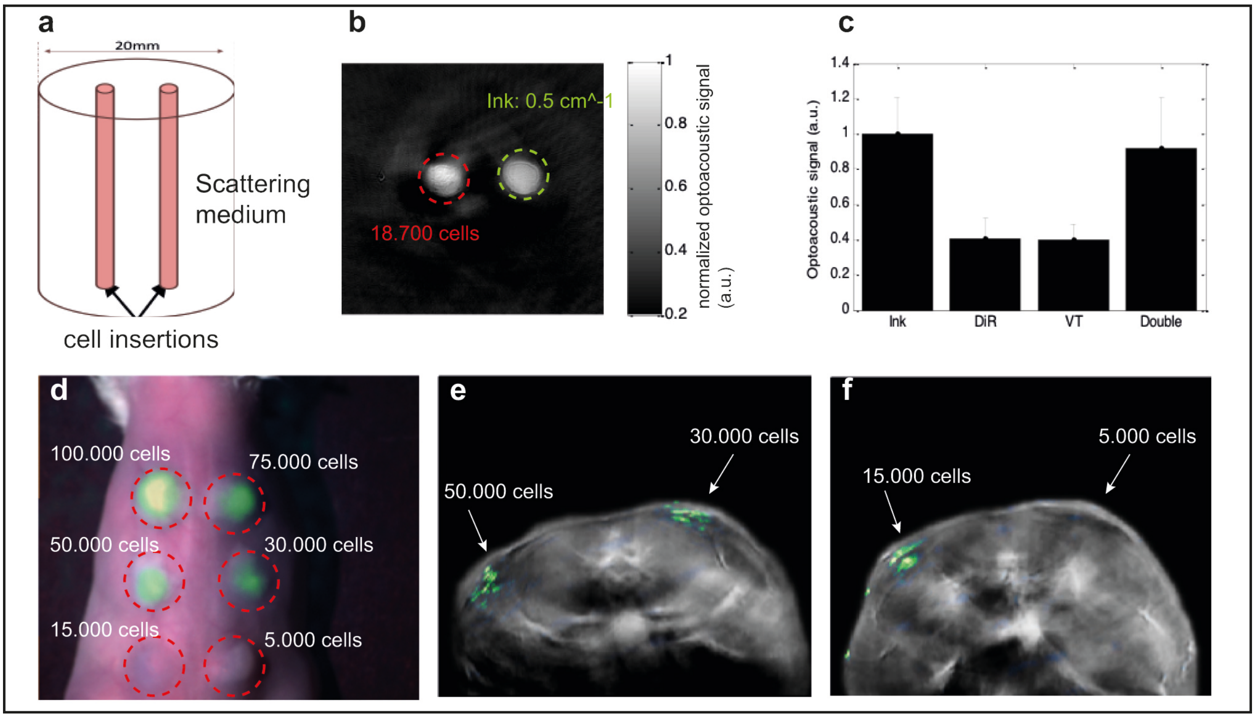

Figure S3. Sensitivity detection of double labeled OT-1 T cells. (**a**) Schematic representation of the phantom used for *in vitro* cell MSOT. (**b**) Optoacoustic image (excitation wav. 760 nm) of double labeled T cells next to a solution of black india ink at a known absorption coefficient. Scale bar 1 cm (**c**) Comparison of the optoacoustic signal produced using India ink of 0.5 cm^{-1} absorbance (760 nm), and 18.700 DiR labeled, VivoTag 750 labeled or double labeled T cells. The bar heights indicate the mean signal over the area imaged and the standard deviations indicate the variation of the optoacoustic signal in this area. (**d-f**) Study assessing the sensitivity of MSOT in detecting T cells in subcutaneous locations: (**d**) Colour picture with epifluorescence overlay (green pseudocolour) of the back of a euthanized CD1 mouse where different concentrations of cells were injected in 50 µl volumes. Detection result using MSOT for 50.000 and 30.000 cells (**e**) and for 15.000 and 5.000 cells (**f**). The sensitivity limit is accordingly 15.000 ,T cells in a volume of 50 µl.

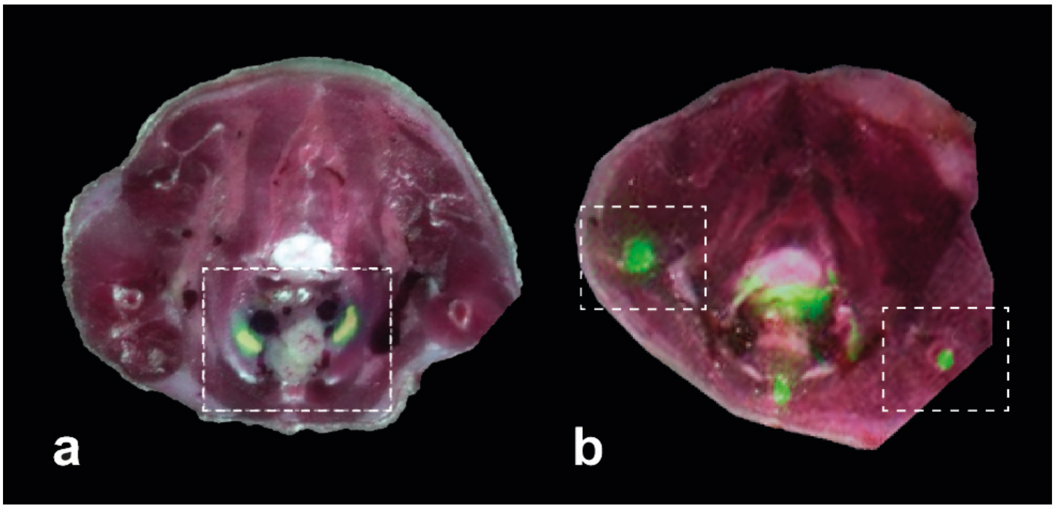

Figure S4. Episcopic fluorescence imaging. Detection of double labeled OT-1 T cells in lymph nodes (**a**) and in the bone marrow (**b**). Cryosection images with overlayed epifluorescence signal from OT-1 T cells (green colour-coding).
